# Ginsenoside Rg3 Suppresses Proliferation and Induces Apoptosis in Human Osteosarcoma

**DOI:** 10.1155/2018/4306579

**Published:** 2018-03-19

**Authors:** Yi Li, Jinying Lu, Furong Bai, Yanan Xiao, Yiran Guo, Ziming Dong

**Affiliations:** ^1^State Key Laboratory of Oral Diseases, Chengdu 610041, China; ^2^School of Basic Medical Science, Zhengzhou University, Zhengzhou 450001, China

## Abstract

Osteosarcoma is the most common primary malignancy of bone in children and the elderly. Recently, more and more researches have demonstrated that Ginsenoside Rg3 (Rg3) is involved in chemotherapy resistance in many cancer, making it a promising Chinese herbal monomer for oncotherapy. In this study, we investigated the efficacy of Rg3 in human osteosarcoma cell lines (MG-63, U-2OS, and SaOS-2). Cell proliferation was measured by CCK8 assay. The migration of cells was examined using the scratch assay method. Quantification of apoptosis was assessed further by flow cytometry. In addition, the expression of apoptosis-related genes (caspase9, caspase3, Bcl2, and Bax) were investigated using RT-PCR. We further investigated the protein level expression of Bcl 2, cleaved-caspase3, and PI3K/AKT/mTOR signaling pathway factors by Western blot assay. Our results revealed that Rg3 inhibited the proliferation and migration of human osteosarcoma cells and induced apoptosis in a concentration- and time-dependent manner. Western blot results showed that Rg3 reduced the protein expression of Bcl2 and PI3K/AKT/mTORbut increased the levels of cleaved-caspase3. Therefore, we hypothesized Rg3 inhibits the proliferation of osteosarcoma cell line and induces their apoptosis by affecting apoptosis-related genes (Bcl2, caspase3) as well as the PI3K/AKT/mTOR signaling pathway. To conclude, Rg3 is a new therapeutic agent against osteosarcoma.

## 1. Introduction

Osteosarcoma is one of the highly primary bone malignancies [1], characterized by immature osteoid formation and abnormal osteoblastic differentiation [2]. It mainly originates from long bones with rapid growth, including the femur, the tibia, and humerus [3]. Osteosarcoma shows a bimodal age distribution with one peak in the young adults and a smaller peak in the elderly [4, 5]. Males are more likely to be affected than females (1.5 : 1). With the application of chemotherapy in the 1970s, 5-year survival rate for patients without metastasis has increased remarkably from 20% to approximate 65%–70% compared with conventional resection surgery previously [6]. Despite the recent progress in osteosarcoma therapy, there have not been any significant improvements in the long-term survival in spite of attempts at comprehensive and intensified treatment [7–9]. Prognosis for patients with recurrence and metastasis (predominantly in the lungs) remains poor [10], due to the high metastatic potential of osteosarcoma cells and lack of biological markers [11]. Hence, it is particularly urgent to explore the mechanisms underlying the occurrence and development of osteosarcoma with the hope of improving the overall survival rate.

**Figure 1:**
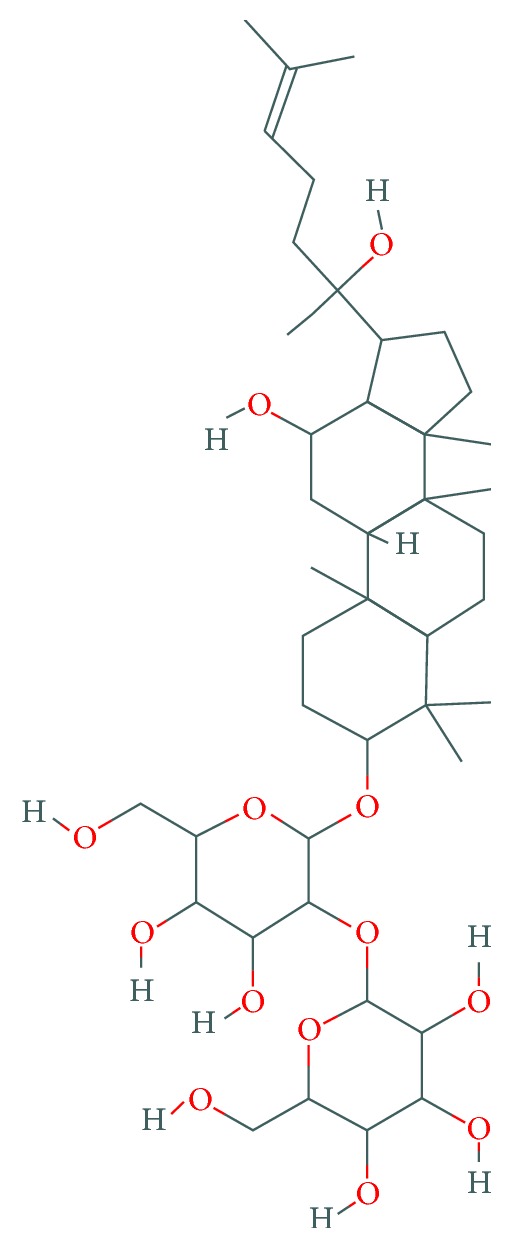
Molecular structure of the ginsenoside Rg3. The image was downloaded from http://www.ncbi.nlm.nih.gov/pccompound.

Ginsenoside Rg3 (Figure 1) is a major active components extracted from a Chinese medical herb, ginseng. It has been reported that ginsenoside Rg3 inhibited the proliferation and migration of various malignant tumors [12, 13]. Previous study found Rg3 could modulate pancreatic cancer cell lines survival via phosphatidylinositol 3-kinase/Akt/mammalian target of rapamycin (PI3K/Akt/mTOR) pathway [14], which play a critical role in many hematological malignancies. Dysregulation of this pathway leads to activation of multiple downstream effectors [15], resulting in uncontrolled cell proliferation and resistance to apoptosis [16].

Thus, we hypothesize that PI3K/Akt/mTOR pathway might be a potential mechanism for the effects of Rg3 on osteosarcoma aggressiveness. We aim to investigate the efficacy of Rg3 on osteosarcoma and attempt to find out the molecular mechanisms for regulatory processes of Rg3 in osteosarcoma to provide new thoughts for the treatment of osteosarcoma.

## 2. Material and Methods

### 2.1. Cell Lines

MG-63, U-2OS, and SaOS-2 obtained from Shanghai Ninth People Hospital were cultured in DMEM medium, supplemented with 10% fetal bovine serum (FBS) (Hyclone, New Zealand), 100 U/ml penicillin (Hyclone, New Zealand), and 100 mg/ml streptomycin (Hyclone, New Zealand) at 37°C in a humidified atmosphere with 5% CO_2_. Rg3 (Meilunbio, China) was prepared with DMSO and diluted to different concentrations with culture medium before treatment. The final DMSO concentration was <0.05%

### 2.2. Cell Counting Kit-8 (CCK-8) Assay

Cell viability was measured using the Cell Counting Kit-8 (CCK8; Dojindo, Japan) assay. The cells were seeded to each 96-well cell culture plate at a density of 4000 cells in 100 *μ*l medium per well. Different doses of Rg3 were added when most cells were attached to the well. After further incubation 24, 48, and 72 h, respectively, at 37°C, 5% CO_2_, 10 *μ*l of the CCK8 reagent was added to each well. Then, after incubation at 37°C for another 1 h, the optical density of each well was measured at 450 nm using a microplate reader (Bio-Rad, USA) and data were analyzed with Microplate Manager 6 to indicate cell viability. All experiments were triplicate.

### 2.3. Cell Scratch Assay

The migration of osteosarcoma cell lines was examined using the scratch assay method. MG-63, U-2OS, and SaoS-2 were seeded into a 24-well plate (1 × 10^5^ cells/well) and incubated with complete medium at 37°C and 5% CO_2_. After 24 h, a scratch in the cell monolayer was created with a sterile 200 *μ*L pipet tip. Cells were washed 3 times with PBS to remove cell debris and then incubated with the FBS-free medium containing different concentrations of Rg3 to eliminate the potential effects of FBS on cell migration. Images of the scratch were acquired using a digital camera (Leica DFC425C, Germany) connected to a phase-contrast microscope (Leica DMIL LED, Germany) at 0 h, 12 h, 24 h, and 48 h. The image information collection was used LAS V3.8.

### 2.4. Quantification of Apoptosis by Flow Cytometry

The apoptosis was measured using annexin V, a protein that binds to phosphatidylserine (PS) residues which are exposed to the surface of the cells early in apoptosis. Cells were seeded at the density of 1 × 10^6^ cells per well with different concentration of Rg3 in 6-well plates. Subsequently, the cells were collected after 12 h, 24 h, and 48 h, respectively, and washed twice with PBS (pH = 7.4). Then, 300 *μ*L of binding buffer was used to resuspend cells, and 5 *μ*L of annexin V-FITC was added. Double-labeling was performed at room temperature for 15 min in dark. 5 *μ*L of propidium iodide (PI) was added for incubation 5 min followed by flow cytometric analysis. Flow cytometry data were acquired on Accunic 6 (BD Biosciences, USA) and analyzed using CFlow software.

### 2.5. RT-PCR and Real-Time PCR

Osteosarcoma cell lines were seeded into 6-well plates and treated with different concentration of Rg3 for 48 h and then collected in the 1.5 mL EP tube, respectively. Total RNA was extracted according to the manufacturer's protocol for Trizol Reagent (Cwbiotech, China). The total RNA concentrations were measured with NanoDrop 2000c spectrophotometer (Thermo Scientific, USA). Synthesis of cDNAs was performed by reverse transcription reactions with 1 *μ*g of total RNA using the GoScript™ Reverse Transcription System (Promega, USA). The primer sequences were as follows: caspase3 forward: 5′-GAAATTGTGGAATTGATGCGTGA-3′, reverse: 5′-CTACAACGATCCCCTCTGAAAAA-3′; caspase9 forward: 5′-CTCAGACCAGAGATTCGCAAAC-3′, reverse: 5′-GCATTTCCCCTCAAACTCTCAA-3′; Bax forward: 5′-CCCGAGAGGTCTTTTTCCGAG-3′, reverse: 5′-CCAGCCCATGATGGTTCTGAT-3′; Bcl2 forward: 5′-GGTGGGGTCATGTGTGTGG-3′, Reverse: 5′-CGGTTCAGGTACTCAGTCATCC-3′; GAPDH forward: 5′-CACCCACTCCTCCACCTTTG-3′, reverse: 5′-CCACCACCCTGTTGCTGTAG-3′. Glyceraldehyde 3-phosphate dehydrogenase (GAPDH) was used to normalize the data to determine the relative expression of the target genes. The qPCR conditions consisted of 95°C for 5 minutes as holding stage, followed by 40 cycles including denaturation at 95°C for 15 seconds and annealing at 60°C for 30 seconds. The relative quantitation of gene expression levels were expressed using the 2^−ΔΔCT^ method. All reactions were carried out in triplicate.

### 2.6. Western Blotting Analysis

MG63, U-2OS, and SaOS-2 cell lines were harvested with different concentration of Rg3 for 48 h, prior to being washed twice with PBS. Cells were lysed using radioimmunoprecipitation assay (RIPA, Sigma, USA) lysis buffer containing 1 *μ*M PMFS (Sigma, USA) for 30 min on ice followed by centrifugation at 12,000 ×g for 5 min at 4°C and supernatants were collected. Total protein concentration was determined using NanoDrop 2000c spectrophotometer (Thermo Scientific, USA). Aliquots of equal protein amounts (200 *μ*g) were separated bySDS-polyacrylamide gel electrophoresis (PAGE) followed by being transferred to nitrocellulose membranes (Millpore, USA). After being blocked in Tris Buffered Saline with Tween-2 (T-BST) with 5% skim milk at room temperature for 1 h, the membranes were incubated with primary antibodies (Abclonal, China) against caspase3 (1 : 1000), cleaved caspase3 (1 : 1000), Bcl2 (1 : 1000), phosphorylated(p)-AKT (Ser473) (1 : 1000), PI3K (1 : 1000), and mTOR (1 : 1000) for overnight at 4°C and then washed three times in TBST. Subsequently the membranes were incubated with corresponding horseradish peroxidase- (HRP-) conjugated secondary antibodies (1 : 2000, Abclonal, China) at room temperature for 1 hour. An enhanced chemiluminescence (ECL) solution (Beyotime, China) was used to visualize the target bands. The protein expression levels were normalized to *β*-actin as an internal control.

### 2.7. Statistical Analysis

Statistical analysis was carried out using an independent sample *t*-test in SPSS 16.0 statistical software, with a *p* value less than 0.05 being considered statistically significant.

## 3. Results

### 3.1. Rg3 Inhibits the Proliferation of Osteosarcoma Cells

**Figure 2:**
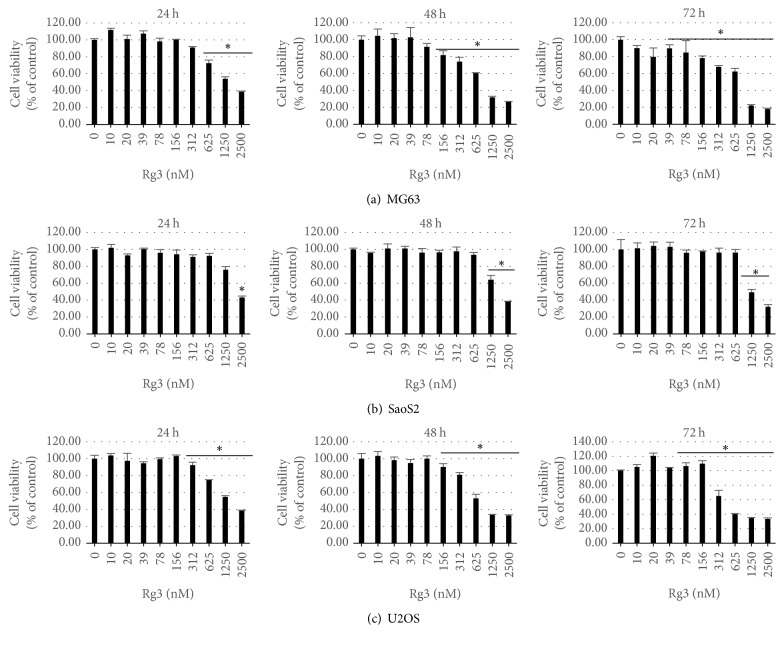
*Rg3 inhibition of human osteosarcoma cells proliferation*. Osteosarcoma cells were incubated with varying concentrations of Rg3 for 24 h, 48 h, and 72 h. Cell viability was determined by CCK-8 assay. Each sample was run in triplicate and was normalized to cells without any treatment. Data represent the mean ± SD. ^*∗*^*p* < 0.05.

The effects of Rg3 on the growth and proliferation of MG-63, U-2OS, and SaOS-2 cells were measured by CCK8 assays. The results shown in Figure 2 demonstrate that Rg3 has a concentration- and time-dependent inhibitory effect on the proliferation of MG-63, U-2OS, and SaOS-2 cells. The concentrations of Rg3 inhibited cell viability with significant effects compared to those of untreated group (*p* < 0.05) were 156 nM for MG-63 cells, 1250 nM for SaOS-2 cells, and 156 nM for U-2 OS at 48 h, respectively. These concentrations were used as median effective concentrations for the following experiments.

### 3.2. Rg3 Treatment Induced Apoptosis of MG-63, U-2OS, and SaOS-2 Cells

**Figure 3:**
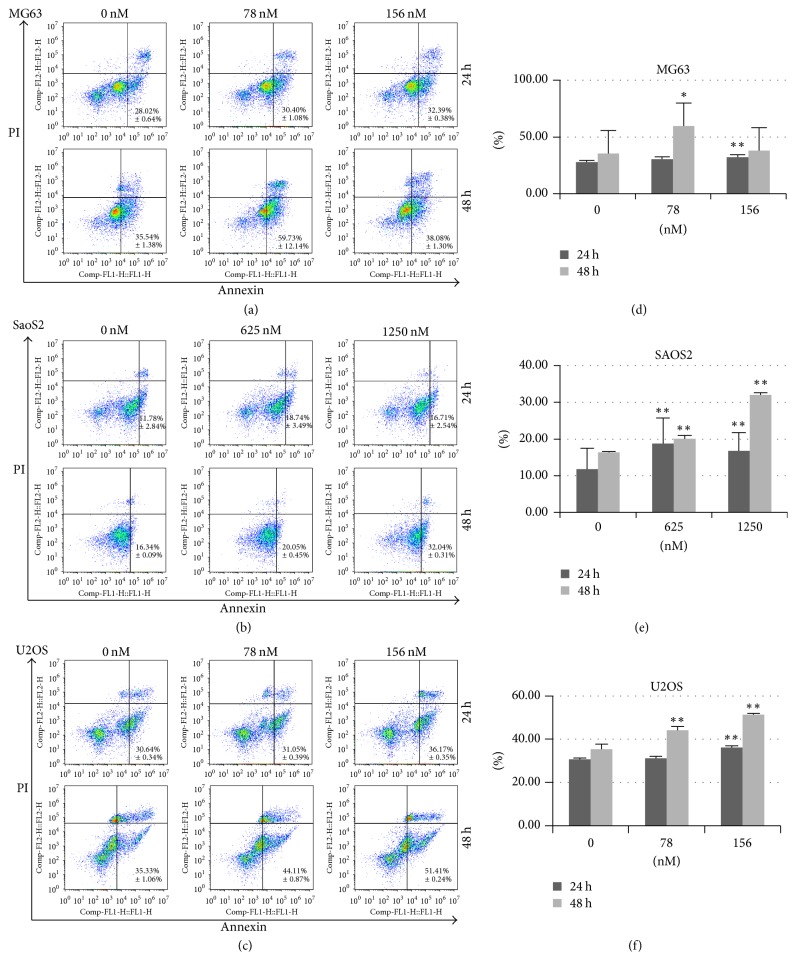
*The effect of Rg3 on apoptosis and necrosis of MG63, SaOS-2, and U-2OS cell lines evaluated by flow cytometry assay*. The cells were double-stained with FITC-Annexin V and propidium iodide (PI). Mean values of the percentage of apoptotic and necrotic cells from four independent experiments ± SD are presented. Percentage of apoptotic cells was the sum of percentage early apoptotic (LR) and late apoptotic cells (UR). ^*∗*^*p* < 0.05; ^*∗∗*^*p* < 0.001.

To investigate the role of Rg3 on the apoptosis of osteosarcoma cell lines, we performed the Annexin V-FITC/PI staining and flow cytometry assay to assess the apoptotic rate of MG-63, U-2OS, and SaOS-2 cells. The percent of apoptotic and necrotic was shown in Figures 3(a), 3(b), and 3(c). As depicted in Figures 3(d), 3(e), and 3(f), after incubation treated with Rg3, the percent of apoptotic osteosarcoma cells increased in comparison to the control cells. It elucidated apoptotic role of Rg3 on osteosarcoma cells.

### 3.3. Rg3 Inhibited the Migration of Osteosarcoma

**Figure 4:**
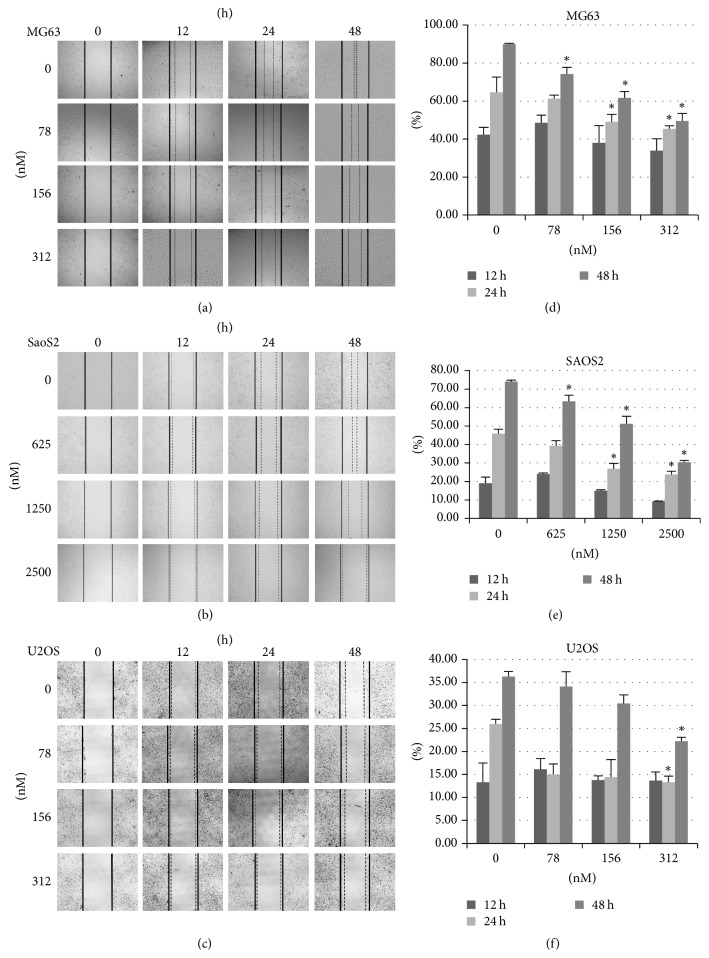
*Rg3 suppression of human osteosarcoma cells migration*. (a–c) Images were captured 0 h, 12 h, 24 h, and 48 h subsequent to the wound being made. (d–f) Cell migration distances were analyzed by SPSS. Data are presented as the mean ± SD. ^*∗*^*p* < 0.05.

An “in vitro” scratch wound healing assay was performed to investigate the effect of the Rg3 on the migration of osteosarcoma cell lines. As shown in Figure 4, we applied different concentration of the Rg3 and observed the migratory condition at 0, 12, 24, and 48 h. The migration rate of the control cell monolayer in MG-63 and SaOS-2 cells was approximately 80% after 48 h incubation, whereas the groups treated with the Rg3 showed significantly decreased migratory ability compared to the control group in both dose- and time-dependent manners. Although it is not obvious, the migration potential of U-2OS cells without treatment was greater than that of Rg3 treated cells. The results indicate that Rg3 inhibits the migration of osteosarcoma cells.

### 3.4. Rg3 Suppressed the Activation of PI3K/AKT/mTOR Pathway in MG-63, U-2OS, and SaOS-2 Cells

**Figure 5:**
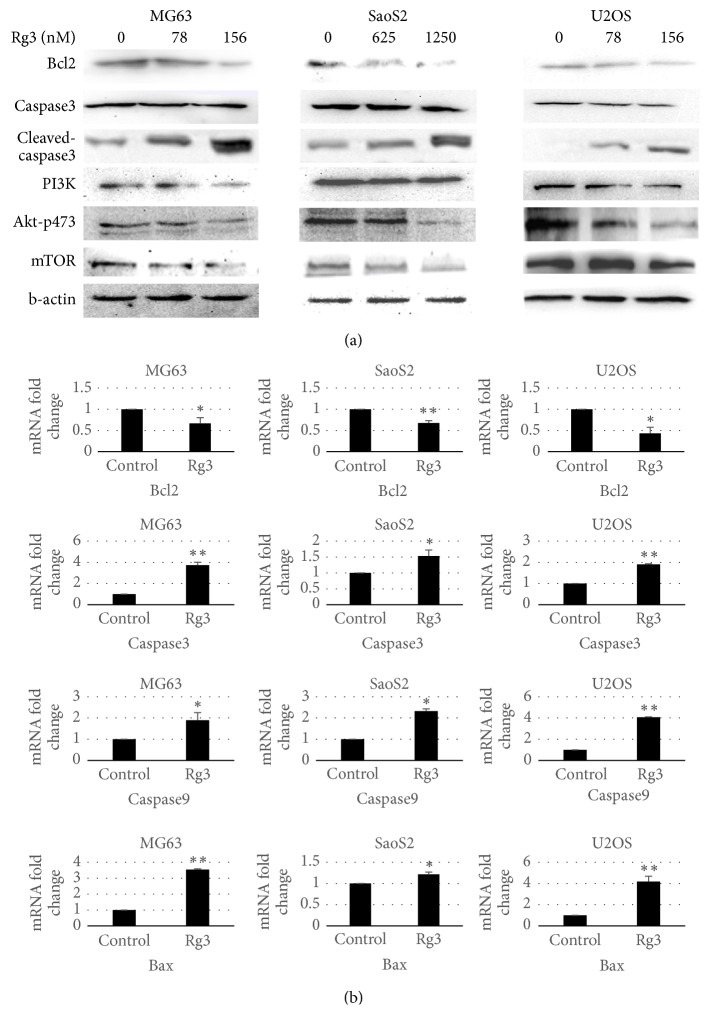
*Effect of Rg3 on Akt phosphorylation and expression levels of Bax, Bcl-2, and caspase in human osteosarcoma cells*. (a) Total protein from whole cell lysates was extracted to analyze Bcl2, caspase 3, PI3K, pAkt, and mTOR assessed by Western blotting. (b) Reverse transcription-quantitative polymerase chain reaction analysis of Bcl2, caspase3, caspase9, and BAX mRNA expression levels in osteosarcoma cells. Data are presented as the mean ± SD. *n* = 6. ^*∗*^*p* < 0.05  ^*∗∗*^*p* < 0.01.

To analyze the intracellular mechanism underlying Rg3 suppressed proliferation and induced apoptosis in MG-63, U-2OS, and SaOS-2 cells, we investigated the involvement of PI3K/AKT/mTOR pathway. As shown in Figure 5(a), a downregulation of PI3K, phosphorylated Akt, and mTOR was observed using Western blot assay after 48 h treatment with Rg3 for all MG63, SaOS-2, and U-2OS cells in a dose-dependent matter. These results demonstrated that Rg3 suppressed proliferation of osteosarcoma cells by inhibiting the PI3K/Akt/mTOR pathway. Bcl2 is the primary markers of cells undergoing apoptosis. Bcl2 expression was measured by PCR (Figure 5(b)) and Western blot (Figure 5(a)) at 48 h later after treatment. The results showed Rg3 treatments downregulated the expression of Bcl2 in MG63, SaOS-2, and U-2OS cells, indicating the occurrence of apoptosis, which is consistent with the previous flow cytometry and PCR results.

### 3.5. Rg3 Induced Osteosarcoma Apoptosis through Bcl2/Bax-Caspase9-Caspase3 Pathway

To confirm the apoptotic effect of ginsenoside Rg3 on three human osteosarcoma cell lines, the expression levels of Bax, Bcl-2, caspase3, and caspase9 were detected by real-time RT-qPCR assay and relative quantification using GAPDH gene transcript as a reference. As shown in Figure 4(b), the mRNA level of apoptosis-related factors (Bax, caspase3, and caspase9) was significantly higher than that in corresponding control cells, while the mRNA expressions of antiapoptotic molecules Bcl2 in three cell lines treated with Rg3 were decreased. We also evaluated caspase3 enzymatic activity using Western blot assay and the results (Figure 5(a)) indicated the presence of Rg3 increased the expression of cleaved-caspase3. These results suggest that the Rg3 could induce apoptosis of MG-63, U-2OS, and SaOS-2 cells dose-dependently through the Bcl2/Bax-caspase9-caspase3 pathway.

## 4. Discussion

Osteosarcoma is the most common primary malignant tumor of bone with high incidence of lung metastasis. The currently standard therapy consists predominantly of surgical resection and postoperative chemotherapy. Despite the development of modern surgical technique and systemic chemotherapy, the prognosis remains poor. Therefore further understanding of the molecular pathogenesis of human osteosarcoma is required in novel therapies.

Ginsenoside Rg3 is the main effective component of ginseng which is an established traditional herbal medicine. At present, ginsenoside Rg3 is mainly used as a tumor angiogenesis inhibitor to prevent the recurrence and metastasis of various malignant tumors after traditional therapy because of its efficacy on the proliferation of tumor vascular endothelial cells and the formation of new blood vessels by regulating some cytokine antiangiogenic factors [17].

This study aims to investigate the effect of ginsenoside Rg3 on osteosarcoma treatment. To observe the effect of Rg3 on apoptosis, we investigated the expression of apoptosis-related genes, such as Bcl2 and caspase families [18]. RT-PCR analysis showed that Rg3 upregulated the expression of Bax and downregulated that of Bcl2. Bcl-2 was the first gene to be identified as an inhibitor of apoptosis, which operates as a checkpoint of upstream caspases [19]. When the expression levels of Bax are increased, Bax can form a homologous dimer with Bcl-2 and activate caspase9 and then cleave the downstream factor procaspase3, finally promoting apoptosis [20, 21]. As an apoptotic markers, caspase3 plays a key role in apoptosis [22]. Therefore, examination of caspase3 activity is the most reliable factor for apoptosis. We then speculated expression of caspase9/caspase3 using RT-PCR. The results demonstrated that Rg3 significantly increased the activation of caspase9/caspase3. Furthermore, Western blot assay illustrated that the protein level of caspase3 was decreased with the cleaved-caspase3 being upregulated, indicating the activation of caspase3. These results indicate that Rg3 induced the apoptosis of osteosarcoma cells through the Bcl-2-caspase9-caspase3 pathway.

Meanwhile we tried to explain the mechanisms whereby Rg3 inhibits proliferation and migration of human osteosarcoma cells and to decipher how Rg3 induces apoptosis. Growing evidence has revealed that PI3K/AKT/mTOR signaling pathway takes part in oncogene activating mutations, oncogene proliferation, and inactivation of cancer suppressor genes in various cancers including osteosarcoma [23–26]. PI3K, a lipid kinase family, transducts signaling proteins to the cellular membrane by generating phospholipids, which then recruit AKT to the cell membrane and phosphorylate it on Ser473. Once phosphorylated and activated, AKT regulates a wide range of biological responses with numerous downstream molecules, such as caspase and mTOR [27]. Activated mTOR contributes to a number of critically important cell processes such as cell growth and proliferation through regulating protein synthesis [21]. PI3K/AKT/mTOR pathway is believed to be involved in the elevation of cell proliferation and suppression of apoptosis [28]. Therefore, we investigated these related protein changes in the Rg3 treated and untreated osteosarcoma cells. The Western blot results showed that, after incubation with different concentrations of Rg3, the expression of PI3K, AKT, and mTOR was significantly decreased in MG-63, U-2OS, and SaOS-2 cells compared with the control group. This could explain the previous results of inhibition of osteosarcoma cell proliferation and induction of apoptosis. Based on these results, we confirmed that Rg3 inhibits proliferation and migration though PI3K/AKT/mTOR pathway.

Akt is very important in BAD (Bcl2-Antagonist of Cell Death) regulation by phosphorylating of either Ser-112 and Ser-136 on BAD to dissociate from Bcl-XL [29, 30], which reverses the death repressor activity Bcl2 family [31] and then affects the expression of downstream signal factor caspase9/caspase3. In addition, Cardone et al. reported active AKT can regulate caspases9 directly by phosphorylating recombinant caspase9 in vitro on serine-196 and inhibiting its protease activity [32]. Therefore the PI3K pathway, in particular AKT, appears to act as a link between cell survival factor receptor signaling and the apoptotic pathways. However, the exact mechanism how Rg3 inhibits the PI3K/AKT/mTOR pathway remains to be fully elucidated.

In conclusion, these data demonstrate that Rg3 can inhibit proliferation and migration and induce apoptosis through PI3K/AKT pathway in osteosarcoma cells. And the dramatic effects of Rg3 in human osteosarcoma cells indicated that Rg3 could be a promising candidate as chemotherapeutic agent against human osteosarcoma.
